# Exploring the origin of the cancer stem cell niche and its role in anti-angiogenic treatment for glioblastoma

**DOI:** 10.3389/fonc.2022.947634

**Published:** 2022-08-25

**Authors:** Funto A. Akindona, Stephen C. Frederico, John C. Hancock, Mark R. Gilbert

**Affiliations:** ^1^ Neuro-Oncology Branch, Center for Cancer Research (CCR), National Cancer Institute (NCI), National Institutes of Health, Bethesda, MD, United States; ^2^ University of Pittsburgh School of Medicine, Pittsburgh, PA, United States; ^3^ Cancer Research UK Cambridge Institute, University of Cambridge, Cambridge, United Kingdom; ^4^ Division of Neurosurgery, Department of Clinical Neurosciences, University of Cambridge, Cambridge, United Kingdom

**Keywords:** CNS, cancer stem cells (CSC), glioblastoma, GBM, CSC

## Abstract

Cancer stem cells are thought to be the main drivers of tumorigenesis for malignancies such as glioblastoma (GBM). They are maintained through a close relationship with the tumor vasculature. Previous literature has well-characterized the components and signaling pathways for maintenance of this stem cell niche, but details on how the niche initially forms are limited. This review discusses development of the nonmalignant neural and hematopoietic stem cell niches in order to draw important parallels to the malignant environment. We then discuss what is known about the cancer stem cell niche, its relationship with angiogenesis, and provide a hypothesis for its development in GBM. A better understanding of the mechanisms of development of the tumor stem cell niche may provide new insights to potentially therapeutically exploit.

## Introduction

Cancer stem cells (CSCs) are a small subset of tumor cells that are thought to be one of the main drivers behind tumorigenesis and cancer propagation (1). This is because of the CSC’s ability to recapitulate the tumor heterogeneity, when isolated and transplanted (2, 3). CSCs accomplish this through self-renewal, in which stem cells create new stem cells *via* asymmetrical or symmetrical division (4). These subpopulations of tumor cells have been described in various liquid and solid tumors including head and neck cancer, breast cancer, and brain tumors (3, 5). There are two primary hypotheses for the emergence of CSCs at tumor initiation: 1) resident healthy tissue stem cells can acquire mutations that results in malignant transformation and 2) healthy somatic tissue cells acquire mutations such that these cells gain stem-like and oncogenic characteristics (6). There is evidence in favor of both explanations in various tumors (6–13). The biomarkers that are used to isolate and identify putative CSC populations vary from cancer to cancer, but among the most common are CD44 and CD133, although some controversy remains (5, 6).

In many cancers, CSC populations associate closely with the vasculature of tumors in what is known as a perivascular niche (PVN) (14–18). The concept of a PVN for CSCs was first described by Calabrese et al. in 2007 in the context of brain tumors (15). Using nestin as a CSC biomarker, they found that nestin-expressing CSCs in glioblastoma (GBM), medulloblastoma, ependymoma, and oligodendroglioma were significantly closer to tumor blood vessels than tumor cells not expressing nestin. Additionally, in medulloblastoma and ependymoma cell culture, CD133-expressing CSCs physically interacted with endothelial cells (ECs) in coculture (15). In-depth exploration and characterization of CSC PVNs, particularly in GBM, has been described in the literature (19–29). GBM PVNs include much more than just CSCs and ECs; pericytes, reactive astrocytes, tumor-associated macrophages and microglia, and fibroblasts are other components that contribute to the maintenance of the GBM CSC PVN (19).

The existence of GBM CSCs (also referred to as glioma stem cells, GSCs) and their PVN are of special importance when considering the aggressive behavior of GBM. GBM is the most common as well as one of the most lethal primary brain tumors, with median overall survival being ~15 months despite standard of care treatment (30). Additionally, despite optimal treatment, within just 1 year of diagnosis approximately 70% of patients with GBM experience recurrence (31). Much of the treatment resistance noted in GBM has been attributed to GSCs which are plastic and can adapt to changes in the tumor microenvironment (TME) to ensure the survival of the tumor (29). These adaptations include the ability to promote tumorigenesis even if a majority of the tumor is removed (28, 29). Given that GSCs are maintained in niches surrounding tumor vasculature and that GBM is a highly vascularized cancer, disrupting tumor angiogenesis was a promising treatment that could target GSC maintenance. Vascular endothelial growth factor (VEGF) is one of the main inducers of angiogenesis and serves as a potential target for treatment (32).

Bevacizumab is a potent humanized antibody against VEGF-A that alters VEGF-A’s binding to ECs, thus downregulating angiogenesis (33). Bevacizumab, as a monotherapy or in conjunction with chemotherapy was thought to have clinical efficacy in patients with recurrent GBM, although subsequent randomized studies failed to demonstrate a survival benefit (34–42). Additionally, pre-clinical models suggest that antiangiogenic therapy causes vascular normalization which results in enhanced blood flow, and therefore enhanced oxygen and drug delivery (43). A landmark phase 3 clinical trial tested whether there was any survival benefit of adding bevacizumab as a first-line treatment in newly diagnosed GBM (41). The trial showed that there was no overall survival benefit with early administration of bevacizumab (41). There was an increase of progression-free survival, however it did not reach predetermined criteria (41). An additional phase 3 clinical trial published in the same year evaluating the survival benefit of bevacizumab in conjunction with lomustine showed similar results (42). These modest results highlight the need to reimagine anti-angiogenic therapy and how to best target tumor vasculature to disrupt GSC maintenance.

The genesis of the GSC niche may provide important insights about GBM tumor biology.

The majority of the publications regarding GSC PVNs focus on characterizing the niche and its components (19–29), but discussion on how the niche develops seems to be a field largely unexplored. In this review, we describe the development of nonmalignant stem cell niches as well as discuss the mechanisms of angiogenesis in cancer. We will also highlight what is known about the CSC niche and how it relates to angiogenesis. Finally, we will provide a hypothesis for the development of CSC niches in GBM.

## Nonmalignant stem cell niches

Many of the pathways involved in the stem cell phenotype of nonmalignant PVNs are also involved in maintaining the CSC phenotype. PVNs have been established in many postnatal tissues including brain, bone marrow, stomach, intestine, and pancreas (44–47). Insight into how these niches form during embryonic development and their maintenance throughout adulthood may help to better understand how CSC niches develop. We discuss the neural stem cell (NSC) niche and the hematopoietic stem cell (HSC) niche for this purpose. There is evidence to support that many brain tumors can arise from the NSC niche (48, 49). It has been shown that NSCs within the SVZ possess GBM driver mutations and have the capacity to migrate from the SVZ and contribute to the development of brain tumors in other regions of the brain (49). Not only do GSC niches express stem cell markers and proteins reminiscent of the NSC niche, but this niche can help drive brain tumor formation by introducing mutations into the ventricular-subventricular zone (V-SVZ) (11, 48, 50–53). SVZ-derived NSCs have also been shown to serve as spatial cues for invading glioma cells, highlighting the complex interplay between the SVZ (54). There is also evidence that GSC niches express stem cell markers and proteins that are mainly found in the HSC niche in the bone marrow (55–57). Exploring the development of NSC and HSC niches could provide valuable insight into GSC niche development.

*Neural Stem Cell Niche*. NSCs are progenitor cells that ultimately differentiate into neurons and glial cells. During embryogenesis, these cells are extremely proliferative but become mostly quiescent once the nervous system is fully developed (58). In the postnatal brain, the V-SVZ and subgranular zone (SGZ) constitute the perivascular niches where NSCs reside. NSCs are heterogenous in nature, and this is thought to be partially due to the difference in the amount of time that it takes these niches to develop (59). In general, vasculature is not necessary for the development of primitive neural networks and in the early embryologic stages of the mammalian brain development (60). In human brain development, the formation of the neural tube occurs during the 3^rd^ and 4^th^ week of gestation (61), while cortical development begins in the 6^th^ week of gestation (62). However, it is not until the 8^th^ week of gestation that neuro-vasculature becomes essential for the developing brain (62). Additionally, as mice age, NSCs in the V-SVZ become more closely associated with brain vasculature (63, 64). This suggests that the role of vasculature in brain development occurs only after initial components of the nervous system and the NSC environment have been established. In contrast, the role of NSCs in the development of the neural vascular network are immediately apparent (65). As the brain develops and grows, simple diffusion is no longer a sufficient method of oxygen delivery for NSCs. NSCs driven by hypoxia/HIF activity express VEGF to serve as a spatial cue for vessel ingression. This finding has been validated throughout the literature as it has been shown that mice with reduced expression of VEGF have decreased vascular density, smaller brains, and portions of their forebrain that are completely avascular (65). In mice with a conditional knockout mutation that ablated NSC function in late embryogenesis, cortical vessel density was greatly reduced and showed significant vessel regression (66). This finding underscores the essential role NSC expression of VEGF has in neurovascular development. In 22 week old human fetal cortical samples, NSCs expressing CXCl2 were shown to have basal processes that exerted a pro-angiogenic effect *via* direct contact with blood vessels (67). This further highlights the importance of NSC’s role in neurovasculature development and patterning.

Additionally, NSCs promote vessel ingression, the incorporation of endothelial progenitor cells into the neural tube, *via* Wnt signaling. NSC-specific Wnt ligands such as Wnt7a and Wnt7b are highly expressed within the neural tube. Decreased expression of these ligands resulted in a reduced number of ECs and pericytes in the neural tube (65). Wnt7a expression may also serve as a spatial cue for ECs as it has been shown to induce EC migration *in vitro (* 65 *)*. After the initial ingression and expansion of blood vessels into the cortex, there is a marked decrease in blood vessel branching frequency. NSCs mediate this stabilization of blood vessel formation *via* the downregulation of endothelial Wnt signaling (65, 68). NSCs also play a role in promoting the integrity and maturation of the neuro-vasculature *via* the expression of integrin αvβ8. This has been evidenced in the literature as it has been shown that mice with either mutated αv or mutated β8 experience severe cerebral hemorrhaging (69, 70). In the postnatal and adult murine brain, the structure of blood vessels as well as their placement within the V-SVZ are quite dissimilar to the neuro-vasculature residing outside neurogenic niches. Like the vasculature in brain tumors (such as GBM), these vessels have sparse pericyte and astrocytic coverage which contributes to these vessels being more permeable or “leaky” as compared to the vasculature in other areas of the brain (58, 60). While a great deal of work has been performed to describe the cytoarchitecture of the adult SVZ in humans (71, 72), there are a paucity of manuscripts describing specifically how the vascular networks are organized in the adult SVZ in humans.

One way the vasculature within the niche maintains the stem-like phenotype of NSCs is through direct contact. NSCs interact with ECs *via* ephrinB2 and Notch ligand JAGGED-1 on ECs which promotes NSC quiescence and stem cell identity (73). More specifically, ephrinB2 suppresses MAPK signaling, JAGGED-1 mediates neural stem cell identity, and they jointly inhibit stem cell differentiation. ECs can also maintain NSC stemness through non-direct contact signaling. These factors/ligands include SDF1, betacellulin (BTC), pigment epithelium-derived factor (PEDF), neutrophin-3 (NT-3), sphingosine-1-phosphate (S1P), and prostaglandin-D2 (PGD2) (58, 74, 75). The interactions between NSCs and ECs is bidirectional as NSCs influence the behavior and development of blood vessels through paracrine and juxtracrine signaling (76, 77). The human NSC line, CTX0E03, highly expresses the well-known proangiogenic factors, VEGFA, epidermal growth factor (EGF), basic fibroblast growth factor (bFGF), angiopoietin 1/2 (ANGPT1/2), transforming growth factor β1(TGFβ1), and hypoxia inducible factor 1α (HIF-1α). When CTX0E03 conditioned media was used, ECs formed tubules when grown in Matrigel. However, when NSCs and ECs were cocultured, which allowed for cell-cell contact, there was a significant increase in EC tubule formation (76). Additionally, vasculature-like structures only formed *in vitro* when undifferentiated NSCs were cocultured with differentiated ECs (77). This finding suggests that the stem-like phenotype of NSCs is necessary for modulation of angiogenesis.

*Hematopoietic Stem Cell Niche.* HSCs are progenitor cells that give rise to all the blood cell lineages. The primary niche for HSCs is in the bone marrow. In embryonic development, hematopoiesis occurs in three waves. The first wave is known as “primitive hematopoiesis” (78). In this short-lived stage, transient hematopoietic cells are created to address the immediate needs of the embryo – oxygenation, tissue defense and repair, and maintenance of circulation (78). In the second wave, known as “definitive hematopoiesis”, erythromyeloid and lymphoid progenitors emerge (78). Erythromyeloid progenitors can then differentiate into mature blood cells. HSCs are generated from the hemogenic endothelium of the aorta-gonad-mesonephros (AGM) in the third wave of development *via* endothelial-to-hematopoietic transition (EHT) (78, 79). It is thought that only a certain subgroup of ECs from the hemogenic endothelium have the capacity to undergo this transition. Signaling pathways such as Notch, Sonic Hedgehog (SHH), and Wnt from the supporting endothelium of the AGM mediate HSC emergence (79, 80). SHH is the primary signaling pathway involved in the formation and specification of the hemogenic endothelium (80). In cell culture assays used to determine promotion of hemogenic endothelium, ECs treated with SHH ligand, Ihh, had higher hemogenic endothelium formation (81). In knockout SHH mutants, activation of hematopoiesis is inhibited, suggesting the crucial role SHH signaling plays in the emergence and regulation of HSCs (82). Notch signaling is required for EHT (80, 83, 84). When activation of Notch signaling is inhibited, there is a marked decrease in conversion of hemogenic ECs to CD45^+^ HSCs in cell culture (83). Additionally, knockout Notch mutants had greatly reduced expression of key factors necessary for hematopoiesis (84). Non-canonical WNT signaling activates the Notch pathway, suggesting that WNT signaling also plays a key role in EHT (84). Indeed, when β-catenin is inhibited before the emergence of HSCs, there is a marked decrease in their generation (84).

In the adult HSC niche of the bone marrow, ECs are the most abundant cell type present as bone marrow vasculature is densely packed. There are two distinct niche types within the bone marrow: the sinusoidal niche and the arteriolar niche (79, 85). Approximately 60% of HSCs reside in the sinusoidal niche (86–88). The sinusoidal vessels are more permeable than the arteriolar ones, so HSCs maintained in this niche have a greater level of reactive oxygen species (ROS) and as such, are more activated (86). Additionally, sinusoidal ECs express higher levels of E-selectin which plays a key role in HSC homing and proliferation (89). Endothelial cytokines such as SCF, CXC12, and JAGGED-1 are also implicated in sinusoidal HSC maintenance, though the relative contributions of SCF and CXC12 from ECs is small in comparison with the contribution from mesenchymal progenitor cells (80, 86, 90). The HSCs maintained in the arteriolar niche are mostly quiescent, due in part to the low levels of ROS which promotes self-renewal. Arteriolar ECs have higher expression levels of vascular cell adhesion molecule-1 (VCAM-1) which has been associated with HSC retention (79, 89). Though these two niches are thought of as distinct, it has been demonstrated that within the sinusoidal niche both quiescent ROS^low^ HSCs and proliferating ROS^high^ HSCs are present (91). The role of the other perivascular cells in the bone marrow niche may have greater impact on the determination of the phenotype of the HSCs.

The influence that HSCs exert on the ECs of the bone marrow vasculature is poorly understood. Studies have shown that HSCs express VEGF-A and can stimulate the *in vitro* proliferation of ECs *via* VEGF (92, 93). Beyond these findings the field remains largely unexplored. In non-malignant stem cell niches, crosstalk between stem cells and niche vasculature is bidirectional. Stem cells are not only maintained by their niche, but also play a role in remodeling the niche to best suit their needs. While there is overlap in the signaling involved in the embryonic development of the two niches, the processes are different. In contrast to HSCs that develop from the endothelium in the niche, ECs must migrate to the NSCs (62, 63, 65, 78, 79).

**Figure 1:**
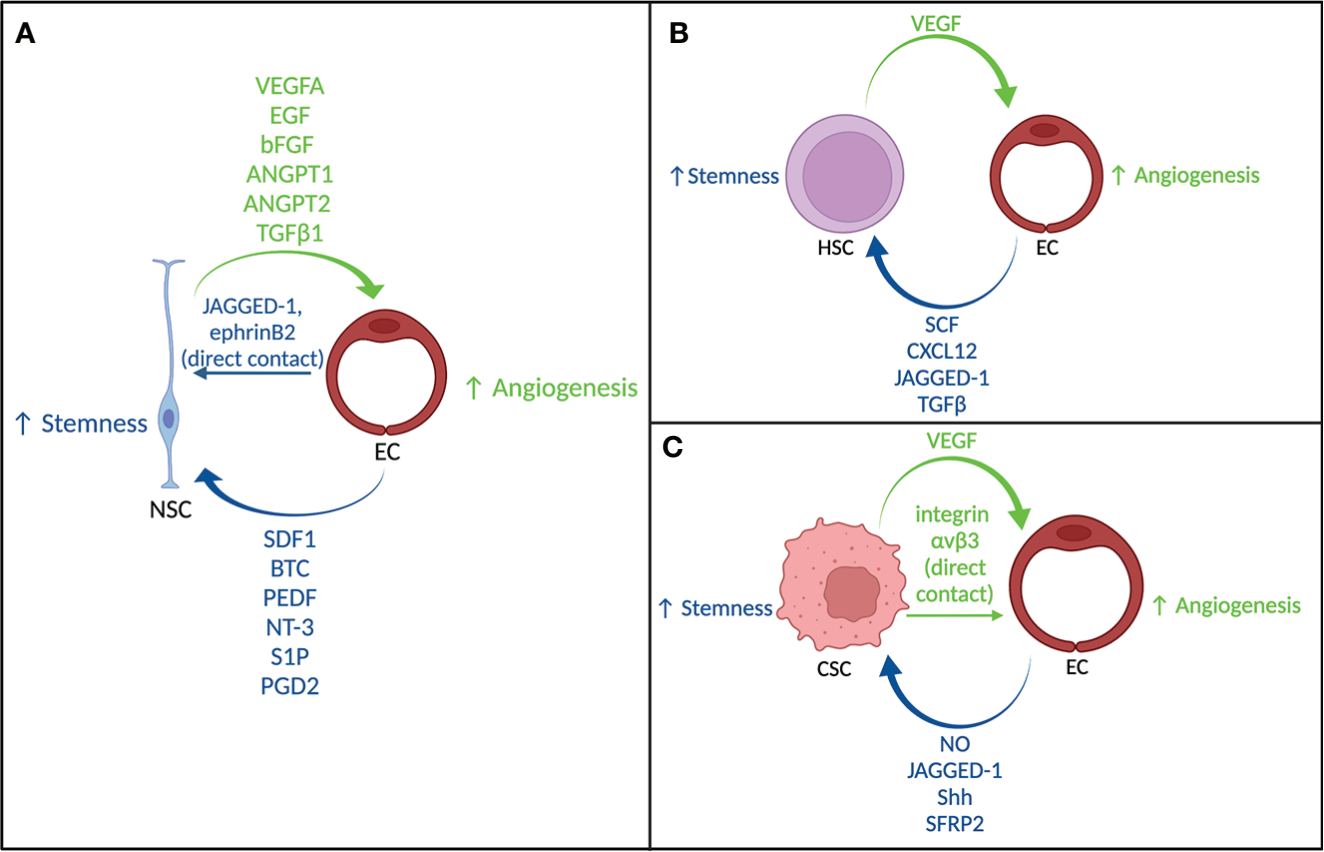
Crosstalk between stem cells and endothelial cells. **(A)** Signaling between neural stem cells and endothelial cells. **(B)** Signaling between hematopoietic stem cells and endothelial cells. **(C)** Signaling between cancer stem cells and endothelial cells.

Considering that cancer stem cell niches display similar signaling and maintenance to these stem cell niche environments, the developmental schemes from these normal systems could inform our understanding of how perivascular niches develop in cancer. **Figure 1** summarizes the key signaling involved in the NSC niche, HSC niche, and CSC niche.

## Angiogenesis and vasculature in cancer

Angiogenesis is an established hallmark of cancer (32). In contrast to normal brain vasculature, tumor vessel morphology and organization tends to be irregular (94). These irregularities include sparse pericyte coverage, weakened interconnectivity between ECs, and irregular basement membrane attachment resulting in leaky vessel networks with inconsistent blood flow (94, 95). Though not to the same extreme, as briefly mentioned in the previous section, the vasculature of the V-SVZ and the sinusoidal vessels of the HSC niche share some similarities with tumor vasculature. This suggests that some aspects of this vessel phenotype prove beneficial for maintaining stem cells and that insights about tumor vasculature development may help to better understand the development of CSC niches. This section will discuss the angiogenic switch in cancer, tumor vascular formation processes, and vessel co-option.

*The Angiogenic Switch.* Whereas homeostatic measures ensure proper function, growth, and organ maintenance, tumorigenesis is thought to begin when cell proliferation becomes unregulated and programmed cell death becomes ineffective. However, these tumors will remain small and dormant if there is a lack of vessel formation. Under angiogenic quiescent conditions, there is a balance between pro-angiogenic factors and anti-angiogenic factors (96). The “angiogenic switch” is flipped when pro-angiogenic signaling dominates (32). This occurs when the tumor reaches a certain size such that simple diffusion of oxygen is not sufficient to sustain tumorigenesis. This low oxygen condition stabilizes HIF1α which leads to the expression of many proangiogenic factors such as VEGF, platelet-derived growth factor (PDGF), placental growth factor (PlGF), ANGPTs, as well as chemokines such as SDF1α and S1P (96, 97). CSCs contribute to neovascularization *via* the expression of pro-angiogenic factors such as VEGF. In GBM, a CD133^+^ cell population, constituting cancer stem cells, express much higher levels of VEGF as compared to CD133^-^ cells (98). Other factors can aid in the induction of the angiogenic switch, including tumor-associated inflammation, as well as infiltration of immune cells (96). The idea of the angiogenic switch/angiogenic dormancy is highly interconnected with tumor dormancy and cancer stem cell dormancy. Tumor dormancy is defined by most of the cells within a tumor remaining viable but not actively proliferating (99). It is thought that CSC niche dormancy, and tumor dormancy by extension, is promoted by angiogenic dormancy. At the time of angiogenic dormancy, the CSC niche may not have the proper signaling pathways in play for activation (100). Once the angiogenic switch is flipped and growth factor signaling can support CSCs, they are released from quiescence and become the main drivers of tumorigenesis (99, 100). The mechanisms mediating this complex relationship are not entirely clear, but it is suggested that CSC niche dormancy is regulated by angiogenic dormancy/angiogenesis (99, 100).

*Vascular Formation Processes.* There are several ways by which tumor vascularization can occur: (1) sprouting angiogenesis, (2) intussusceptive angiogenesis, (3) vasculogenesis, (4) trans-differentiation of CSCs, and (5) vascular mimicry (96). Sprouting results in the formation of a new blood vessel that branches off of a parent vessel. Crosstalk between VEGF and Dll4/Notch signaling mediates this process (101, 102). Intussusceptive angiogenesis occurs when a transluminal epithelium begins to form inside of another blood vessel. This type of angiogenesis has been observed in various cancers including breast, lung, and brain cancer (103–105). While the mechanisms underlying intussusceptive angiogenesis are not clear, VEGF, PDGF, and erythropoietin are involved in its induction (106, 107). This type of angiogenesis is thought to aid in tumor development by increasing the complexity and number of microvascular structures (96). Vasculogenesis involves the *de novo* formation of blood vessels. This process relies on the recruitment of endothelial progenitor cells (EPCs). VEGF signaling results in the mobilization of VEGFR2^+^ bone marrow EPCs to the tumor (108). Additionally, tumor cells secrete various chemokines such as SDF-1, CCL2, CCL5, and adiponectin which mediate the homing of endothelial progenitor cells into tumor neovasculature (109, 110). Additionally, junctional adhesion molecule-C (JAM-C) is also important in EPC homing and vascular assembly (111).

As VEGF is heavily involved in the angiogenic mechanisms, CSCs may play a regulatory role in each. The concept of trans-differentiation of CSCs has been described for several cancers (23, 112, 113). In GBM, it has been observed that a subpopulation of ECs possesses the same somatic mutations found in tumor cells, such as *EGFR* amplification and chromosome 7 alterations (114). Another study demonstrated that GBM CSCs give rise to vascular pericytes (23). Though vascular mimicry is considered a type of tumor vessel formation, this process does not require the contribution of ECs. This has been seen in several tumors and can contribute to tumor development by promoting tumor cell motility and invasion and by providing an alternate route of neovascularization following anti-angiogenic therapy (96). It is not entirely clear how large of a role vascular mimicry plays in tumorigenesis. Whether CSC niches can form around these makeshift vessels is an area that remains largely unexplored.

*Vessel Co-option.* Vessel co-option is a non-angiogenic, and VEGF-independent process in which tumors hijack the vasculature of normal tissue to meet their metabolic needs (115). Invasion of the tumor into surrounding healthy parenchyma is, in part, mediated by vessel co-option (116). Tumor vessel co-option has been previously described in brain tumors, as well as tumors of the liver, lung, and breast (115). Co-option can be determined *via* histopathology as tumor vessels are observed preserving the expression of factors associated with normal vasculature. In non-small cell lung cancer, the alveolar tumor growth pattern retained the normal markers associated with alveolar capillaries indicating that this growth pattern relies on alveolar capillary co-option (117). Similarly, gliomas can possess areas where vasculature still reflects the characteristics of an intact blood-brain barrier, indicative of vessel co-option (118). Normal ECs may serve as a spatial cue for CSCs. In 3D models of GBM, it was shown that normal brain ECs promote tumor growth and invasion *via* an IL-8-dependent pathway that promotes stemness (119).

Tumor vascularization is dynamic, meaning that tumors can rely on vessel co-option or angiogenesis alone, switching as needed, or even have both processes occurring simultaneously in different parts of the tumor (116). Vessel co-option has also been implicated in the resistance of tumors to anti-angiogenic therapy (115). In orthotopic models of glioma and brain metastasis, anti-angiogenic therapy resulted in a more infiltrative phenotype (120–122). In hepatic cancer, the population of patients that responded well to bevacizumab were enriched for more angiogenic-reliant tumors, while those who had poor responses were enriched for more co-option-reliant tumors (123).

## The cancer stem cell niche

A perivascular CSC niche has been described for several different cancers, particularly in brain tumors (15, 100, 124, 125). In much the same way that crosstalk between the nonmalignant stem cells and the vasculature in the niche is bidirectional, the same is true for CSCs in their niche. As discussed previously, one of the major contributions of CSCs to ECs is the expression of VEGF to promote angiogenesis (98). CSCs can also maintain ECs *via* direct contact. In GBM, CSC-EC contact *via* integrin αvβ3 resulted in EC activation (126). This activation is shown *via* an increase in E-selectin and VCAM-1, as well as promotion of EC network formation and a more migratory phenotype (126). Conversely, many of the pathways involved in nonmalignant stem cell maintenance are also involved in the CSC phenotype maintenance. As mentioned earlier, Notch, SHH, and Wnt signaling are crucial for the development and maintenance of nonmalignant stem cells, and the same is true for their involvement with CSCs. In GBM, ECs release nitrous oxide (NO) which activates notch signaling, allowing for a promotion of CSC self-renewal (5, 21). However, in other cancers, alternate mechanisms have been found. For example, in colorectal cancer notch paracrine signaling *via* a soluble form of endothelial JAGGED-1 maintains CSC stemness (127). In breast cancer, ECs activate notch signaling *via* direct contact with CSCs (18). In terms of SHH signaling, in GBM, CSCs were found to closely associate with Sonic hedgehog (Shh)-expressing ECs and inhibition of the SHH pathway *via* Shh knockdown hampered the stem-like phenotype of the GSCs (128). Notch and SHH signaling are downstream of the Wnt signaling cascade (129), signifying the key role Wnt signaling plays in CSC self-renewal and differentiation. Interestingly, endothelial regulation of Wnt signaling *via* secreted frizzled-related protein 2 (SFRP2) in cancer is context-dependent. In brain tumors, breast cancer, ovarian cancer, gastric cancer, and esophageal cancer, the SFRP2 promoter is hypermethylated, suggesting SFRP2 may act as a tumor suppressor (130). However, in osteosarcoma, multiple myeloma, and colorectal cancer, SFRP2 is overexpressed (130). This overexpression of SFRP2 has been associated with poor clinical outcomes (130). While SFRP2 is just one of many methods in which Wnt signaling can be regulated, this protein highlights an important endothelial mediator of Wnt which has important effects on CSC phenotype. Though the mechanism by which ECs maintain CSCs is quite complex, endothelial signaling is necessary (131).

## The developmental hypothesis for the GBM CSC niche

**Figure 2:**
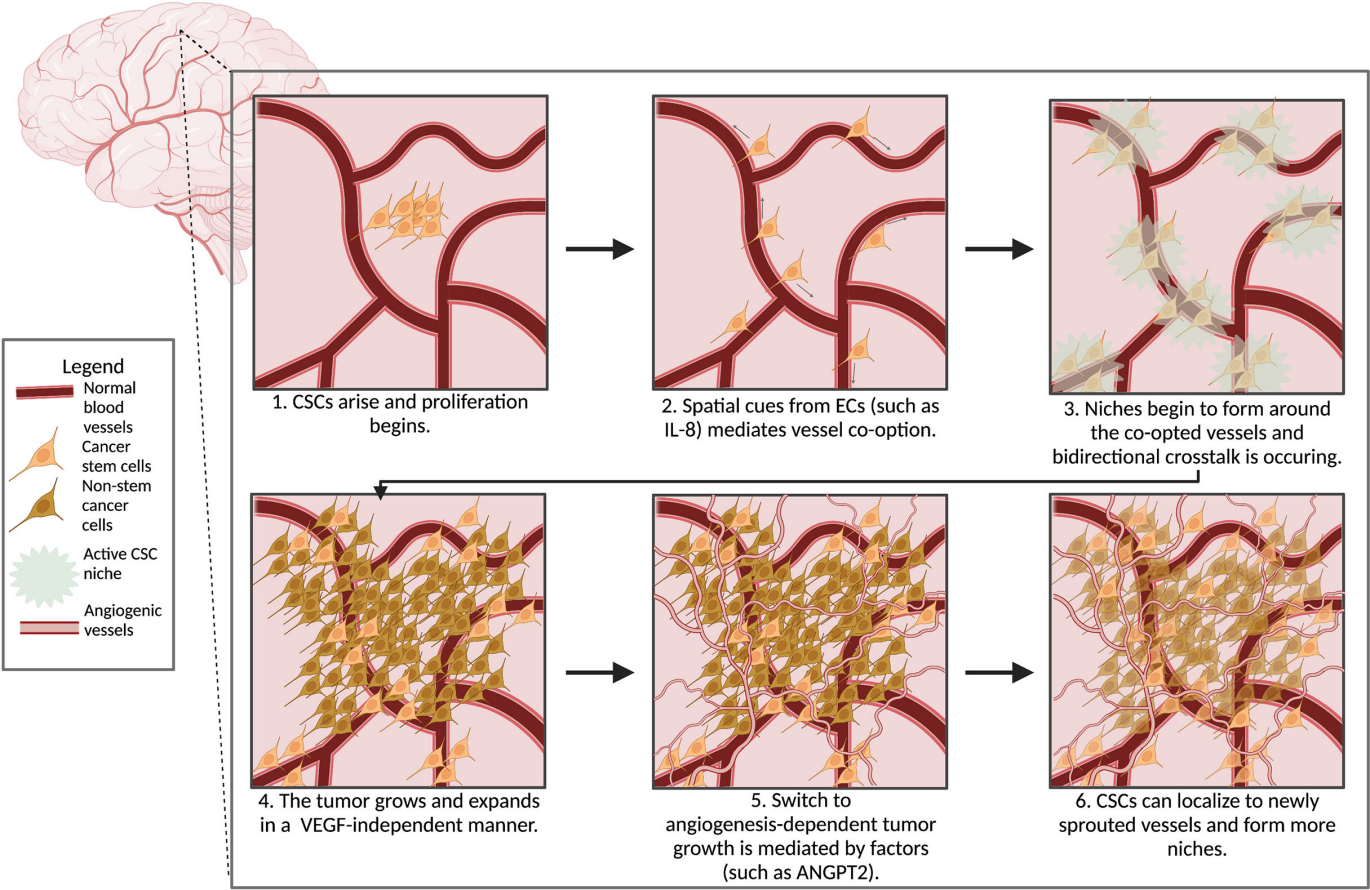
Scheme for the development of GBM CSC niche. Objects are not to scale.

There have been extensive efforts to characterize the GBM CSC niche, however, less is known about the mechanisms that contribute to perivascular niche formation. As described above, angiogenesis is a hallmark of cancer and the CSC niche is thought to be released from dormancy after the angiogenic switch has been activated, suggesting that angiogenesis is a key factor in development of the GBM CSC niche (100). However, in the early stages of gliomagenesis, vessel co-option is the dominant mechanism of tumor vascularization (116, 132, 133). In an orthotopic murine glioma model, it was observed that once the implanted tumor began tissue invasion, the patterning of the tumor overlapped with the pre-existing vasculature of the brain. This finding indicates that healthy brain vasculature was being used as scaffolding for these invading tumor cells, confirmed with a *de novo* murine GBM model and human GBM tissue samples (132). Additionally, a computational simulation of brain tumor development revealed that VEGF-independent vessel co-option is sufficient to sustain tumor growth (132). Another study showed that this VEGF-independent co-option process is mediated by CSCs expressing NSC markers nestin and musashi-1 (133). In the rat C6 glioma model, there was a rapid invasion of tumor cells *via* vessel co-option followed by an upregulation of ANGPT2 (134). The expression of ANGPT2 was associated with vessel regression and hypoxia, thus leading to the induction of VEGF and a rapid expansion of angiogenesis (134). Therefore, while gliomas may initially be VEGF-independent, the tumor likely switches to become an angiogenic-dependent tumor, making angiogenesis a component of CSC maintenance, but likely not driving initial CSC niche formation. As outlined in **Figure 2**, we propose that CSC niches first develop around co-opted vessels and that the beginning of GBM development is angiogenesis-independent. This idea conflicts with the notion of tumor dormancy that was previously discussed. Activation of the CSC niche during vessel co-option could, however, contribute to the release of the angiogenic switch. The cell of origin for GBM CSCs is unknown; this hypothesis does not address whether the CSC arises from a NSC that acquired the requisite mutations to become malignant or is a result of the dedifferentiation of neoplastic brain tissue to a more stem-like phenotype. Our hypothesis regarding the development of CSC niches may provide a framework for additional investigation into gliomagenesis and help better understand treatment resistance, most notably anti-angiogenic therapies.

## Conclusion

In this review we propose a methodology for the development of the GBM CSC niche, hypothesizing that mechanisms of the development and maintenance of the NSC and HSC niches may provide insights. In this context, the role of angiogenesis, a hallmark of GBM was discussed as a potential component of CSC niche development. Though GBM is a highly vascularized tumor, angiogenic-focused treatments have been largely unsuccessful (115). GBM can circumvent anti-VEGF therapies through VEGF-independent means, such as tissue invasion *via* vessel co-option. Targeting vessel co-option may prove to be an effective strategy for targeting tumor vascularization, perhaps in conjunction with anti-VEGF therapy. Factors such as bradykinin, SDF-1α, ANGPT2, IL-8, mammary-derived growth inhibitor (MDGI), inositol-requiring enzyme (IRE)-1α, ephrin-B2, Olig2, and Wnt7a have been shown to be important for vessel co-option specifically in GBM (135). Although outside the scope of this review, the interplay between GBM vessel co-option and the tumor microenvironment, including tumor immune cell populations, remains to be further elucidated. In addition to alternate therapeutic strategies targeting vascular interactions with CSCs, clarifying the mechanisms behind niche formation may reveal additional vulnerabilities that can be exploited with future therapies.

## Author contributions

FA performed a review of the literature, wrote the manuscript, and is the first author.

SF and JH provided their expertise on the clinical aspects of the manuscript. MG conceptualized and supervised the writing of the manuscript. All authors contributed to the article and approved the submitted version.

## Acknowledgments

This research was supported by the NIH Undergraduate Scholarship Program, the Intramural Research Program of the NIH, National Cancer Institute, Center for Cancer Research, and the NIH Oxford-Cambridge Scholars Program. All figures created with BioRender.com.

## Conflict of interest

The authors declare that the research was conducted in the absence of any commercial or financial relationships that could be construed as a potential conflict of interest.

## Publisher’s note

All claims expressed in this article are solely those of the authors and do not necessarily represent those of their affiliated organizations, or those of the publisher, the editors and the reviewers. Any product that may be evaluated in this article, or claim that may be made by its manufacturer, is not guaranteed or endorsed by the publisher.

## Abbreviations

VEGF, vascular endothelial growth factor; PDGF, platelet-derived growth factor; EGF, epidermal growth factor; PlGF, placental growth factor; ANGPT, angiopoietin; SDF1, stromal cell-derived factor 1; S1P, sphingosine-1-phosphate; BTC, betacellulin; PEDF, pigment epithelium-derived factor; NT-3, neutrophin-3; PGD2, prostaglandin-D2; bFGF, basic fibroblast growth factor; TGFβ1, transforming growth factor β1; HIF-1α, hypoxia inducible factor 1α; SCF, stem cell factor; CXCL12, C-X-C Motif Chemokine Ligand 12; NO, nitric oxide; Shh, sonic hedgehog; SFRP2, secreted frizzled-related protein 2; CSC, cancer stem cells; GBM, glioblastoma multiforme; HSC, hematopoietic stem cells; NSC, neural stem cells; GSC, glioma stem cell; MDGI, Mammary-derived growth inhibitor; IRE-1α, Inositol-requiring enzyme-1α; TME, tumor microenvironment.
